# The Transcription Factor MEF2C Negatively Controls Angiogenic Sprouting of Endothelial Cells Depending on Oxygen

**DOI:** 10.1371/journal.pone.0101521

**Published:** 2014-07-02

**Authors:** Caterina Sturtzel, Julia Testori, Bernhard Schweighofer, Martin Bilban, Erhard Hofer

**Affiliations:** 1 Department of Vascular Biology and Thrombosis Research, Center for Physiology and Pharmacology, Medical University of Vienna, Vienna, Austria; 2 Department of Laboratory Medicine, Medical University of Vienna, Vienna, Austria; Medical University Innsbruck, Austria

## Abstract

The MADS box transcription factor MEF2C has been detected by us to be upregulated by the angiogenic factors VEGF-A and bFGF in endothelial cells. We have here investigated its potential role for angiogenesis. MEF2C was surprisingly found to strongly inhibit angiogenic sprouting, whereas a dominant negative mutant rather induced sprouting. The factor mainly affected migratory processes of endothelial cells, but not proliferation. In gene profiling experiments we delineated the alpha-2-macroglobulin gene to be highly upregulated by MEF2C. Further data confirmed that MEF2C in endothelial cells indeed induces alpha-2-macroglobulin mRNA as well as the secretion of alpha-2-macroglobulin and that conditioned supernatants of cells overexpressing MEF2C inhibit sprouting. Alpha-2-macroglobulin mediates, at least to a large extent, the inhibitory effects of MEF2C as is shown by knockdown of alpha-2-macroglobulin mRNA by lentiviral shRNA expression which reduces the inhibitory effect. However, under hypoxic conditions the VEGF-A/bFGF-mediated upregulation of MEF2C is reduced and the production of alpha-2-macroglobulin largely abolished. Taken together, this suggests that the MEF2C/alpha-2-macroglobulin axis functions in endothelial cells as a negative feed-back mechanism that adapts sprouting activity to the oxygen concentration thus diminishing inappropriate and excess angiogenesis.

## Introduction

Angiogenesis, the new formation of blood vessels by sprouting of vessel wall endothelial cells, is primarily induced by vascular endothelial growth factor A (VEGF-A) [1]. When tissue oxygen concentration drops below a metabolically tolerable threshold, hypoxia inducible factors become stabilized in the hypoxic cells and initiate transcription of the VEGF-A gene leading to secretion of the factor [2]. Upon diffusion and binding of VEGF-A to its receptor VEGF receptor 2 (VEGFR2) on endothelial cells in neighboring vessels these start to form sprouts. Tip cells migrate out of the vessel wall followed by proliferating stalk cells eventually forming a new capillary [3]. VEGF-A/VEGFR2 signaling has been shown to strongly activate the PKC/MAPK and Ca^++^/NFAT pathways via PLC-gamma as well as the PI3K/AKT pathway to induce migration, proliferation and survival [4], [5].

Although these initial signaling pathways triggered by VEGF-A/VEGFR2 are well established, we are still devoid of detailed knowledge about the further downstream events leading to the consecutive upregulation of genes essential for the control of sprouting angiogenesis. To further dissect these gene regulatory events we and other groups have performed gene profiling to detect genes specifically induced by VEGF-A [6], [7]. Focusing on transcriptional regulators we have defined four transcription factors as the most strongly and specifically upregulated by VEGF-A, and in part by basic fibroblast growth factor (bFGF), namely nuclear receptor subfamily 4, group A, member 2 (*NR4A2*), early growth response 3 (*EGR3*), H2.0-like homeobox (*HLX*) and myocyte enhancing factor 2C (*MEF2C*). These factors were not inducible by inflammatory mediators. Meanwhile, NR4A2 and EGR3 have been both reported to possess pro-angiogenic functions [8], [9]. Furthermore, in the case of the homeobox transcription factor HLX, we have been able to delineate an important function in the negative control of sprouting which is in part mediated via the HLX-mediated upregulation of the negative guidance receptor UNC5B under normoxic conditions [10].

Moreover, our data had shown that MEF2C is another factor specifically induced by VEGF-A and also bFGF, implicating MEF2C also in the control of angiogenesis [6], [11]. An important angiogenesis-related function of MEF2C was further corroborated by its demonstrated expression in tip cells [12], however its mechanism of action in endothelial cells during angiogenesis has not yet been fully determined. Based on the findings that this MADS box containing transcription factor fulfills important functions in many differentiation processes such as in muscle cell differentiation and is known to be a critical switch in the specification of many cell types including neuronal and lymphatic cells, a corresponding important role in endothelial cells could be anticipated. Additionally, Mef2c-/- mice are embryonically lethal due to prominent heart defects and importantly also display a vascular phenotype characterized by a failure of organization of endothelial cells into an ordered pattern of the vascular plexus [13].

We have therefore here further explored the function of MEF2C and its potential downstream target genes in endothelial cells. MEF2C gain-of-function experiments revealed an inhibitive effect on sprouting suggesting a negative feedback mechanism involving the MEF2C-mediated upregulation of alpha-2-macroglobulin (*A2M*), which is shown by us to reduce angiogenic sprouting. Since *MEF2C* and *A2M* are mainly induced during normoxia, but are not or much less inducible under hypoxia, the data suggest that the MEF2C/A2M axis serves as an angiogenic break under normoxic conditions to adapt new sprout formation to tissue oxygen levels.

## Materials and Methods

### Ethical standards

The authors declare that all experiments performed in this work comply with the current laws of Austria. Human umbilical cords and cord blood samples were obtained at birth after full-term delivery from the Department of Obstetrics and Gynecology of the University Hospital Vienna according a written informed consent procedure and an approval of the local ethical committee of the University Hospital Vienna. This approval (EK 122/2010) was obtained specifically for the FWF project funding this study (P21291-B11).

### Cell culture and materials

Primary human umbilical vein endothelial cells (HUVEC) were isolated as described previously [14]. HUVEC were cultured on 1% gelatine-coated plates in EGM-2 MV medium (Lonza, Walkersville, USA) and used for experiments from passage 2 to 5. Endothelial colony forming cells (ECFC) were isolated similar to described procedures [15] from human umbilical cord blood samples by plating the mononuclear cell fraction in EGM-2 MV medium on rat tail collagen (BD, New Jersey, USA) coated tissue culture plates. The next day floating cells were washed away and residual adherent cells continuously fed every three days with fresh medium for 2–4 weeks, until typical colonies of cobblestone shaped cells became visible. These were further passaged and used for experiments from passage 3 to 6.

HEK293 cells (CRL-1573; ATCC) were grown in minimal essential medium alpha (MEM alpha) completed with 10% newborn calf serum (NCS) and 1% antibiotic mix (all from PAA/GE healthcare, Pasching, Austria). 293T cells (CRL-11268; ATCC) were maintained in Dulbecco's modified Eagle medium (DMEM) (Lonza) with 10% fetal calf serum (FCS; Sigma-Aldrich, Steinheim, Germany).

Cells were grown under normoxic conditions at 37°C, 5% CO_2_ and 21% O_2_ in a Napco cell culture incubator equipped with an oxygen measuring device (Thermo Fisher, Vienna, Austria). “Hypoxia” conditions were applied by flow through of nitrogen reducing oxygen concentration to 1.5%.

Alpha-2-macroglobulin, DAPT and Trichostatin A (all Sigma-Aldrich), VEGF-A and bFGF (both Immunotools, Friesoythe, Germany) were applied in indicated concentrations.

### Generation of recombinant adenoviruses

The cDNA clone of human MEF2C (IRATp970F1023D; http://www.imagenes-bio.de/) was obtained from RZPD, Germany. The coding region from position -530 bp in regard of the ATG initiation codon to position 1413 was subcloned into the *Not*I and *Spe*I restriction enzyme cleavage sites of the multiple cloning site of the pShuttle-IRES-hrGFP-1 vector plasmid, which is part of the AdEasyTM Adenoviral Vector System kit from Stratagene (Agilent Genomics, La Jolla, CA). pShuttle.MEF2C and the pAdEasy-1 were co-transformed into supplied BJ5183 RecA+ E.coli for recombination. The successfully recombined vector was linearized with PacI (New England Biolabs) and transfected into HEK293 cells using a mammalian transfection kit (Stratagene) to generate primary adenoviruses. These were subcloned, amplified and purified by ultracentrifugation over a CsCl gradient (Sigma-Aldrich). An empty control adenovirus was prepared in parallel. Viral titer was determined using the Adeno-X rapid titer kit (Clontech, Mountain View, CA). The dominant-negative MEF2 encoding adenovirus was a generous gift of Dr. Leon de Windt (University of Maastricht, Maastricht, The Netherlands).

### Production of recombinant lentiviruses

Five plasmids (Mission shRNA pLKO.1-puro, Sigma Aldrich, Hamburg, Germany) encoding the corresponding short hairpin RNA (shRNA) sequences targeted against *A2M* and a scattered sequence control shRNA plasmid were purchased from Sigma-Aldrich. The shRNA plasmids were transfected into 293T cells together with 2 packaging vectors (pMD2.G and psPAX.1) using the calcium phosphate method as described [16]. Supernatants were harvested after 24 and 48 h and filtered through a 0.4 µm membrane. For downmodulation of *A2M* mRNA virus preparations for 3 different *A2M* shRNA sequences, individually tested and selected for inhibition of *A2M* mRNA, were mixed at a 1∶1∶1 ratio. For infection the mixed virus supernatants were added to subconfluent ECs together with fresh medium in a ratio of 1∶2.

### RNA preparation and cDNA synthesis

For growth factor stimulation, HUVECs were grown to density, serum-starved overnight using 0.5% FCS in 1 ml EBM medium, and then treated for the indicated time points. For overexpression experiments HUVECs were infected in sub-confluent state with control (Ad.con), MEF2C (Ad.MEF2C) or dominant-negative MEF2 (Ad.dnMEF2) enconding adenovirus with multiplicities of infections (MOI) of 10 to 30 for the indicated time points. For harvesting cells were pretreated with RNAlater (Ambion/Life technologies, Vienna, Austria) for 1 min and then lysed with QIAzol (Qiagen, Maryland, USA). Total RNA was extracted following the manufacturer's protocol. Total RNA (1 µg) was used to synthesize cDNA with RevertAid H Minus Reverse Transcriptase provided with oligo dT18 primers, dNTPs, and RiboLock RNase Inhibitor (all from Fermentas/Thermo Fisher Scientific, St.Leon-Roth, Germany). Procedures were as given by the Fermentas manual.

### Real-time RT-PCR

mRNA levels were measured using real-time reverse transcription-polymerase chain reaction (RT-PCR) detecting SYBR Green I with a Rotor-Gene Q cycler (Qiagen). 1∶2 dilutions of cDNA were analysed with Rotor Gene SYBR Green kit (Qiagen). Values were normalized to beta 2-microglobulin mRNA levels as internal control. Using Primer3 software (http://frodo.wi.mit.edu/primer3/) oligonucleotide primers were designed and used forward/reverse as

MEF2C CATCCACTGCCACCATCTGC/CGTGTGTTGTGGGTATCTCG

A2M ATGTGAGCCGGACAGAAGTC/CTGGGACATCTTGCAGAACC

beta2M GATGAGTATGCCTGCCGTGTG/CAATCCAAATGCGGCATCT

### Affymetrix microarray analysis

Subsequent to infection with Ad.MEF2C and Ad.con for the indicated time points HUVEC were harvested as described under „RNA preparation“. Extracted RNA was further purified using RNeasy kit (Qiagen). Total RNA (200 ng) was analysed on genome-wide human Gene Level 1.0 ST GeneChips (Affymetrix, Santa Clara, CA, USA) as decribed in detail in Tauber S et al. [17]. Scannings of the arrays were carried out according to manufacturer's protocols https://www.affymetrix.com. RMA signal extraction, normalization and filtering was performed as described (http://www.bioconductor.org/). A variation filter was applied for selecting informative (i.e., significantly varying) genes. The filtering criteria for the exemplary data sets required an interquantile range >0.5 and at least one sample with expression intensity >50. The full obtained data sets are now available at Gene Expression Omnibus under the accession number GSE46279 (http://www.ncbi.nlm.nih.gov/geo/query/acc.cgi?acc=GSE46279).

### ELISA analysis

To obtain supernatants to be tested for A2M content, sub-confluent HUVEC in 6-well plates were infected with Ad.con or Ad.MEF2C with MOIs of 3-10 or left uninfected. Cells were further cultured to density in EGM2-MV (Lonza) for 16 h. Then medium was changed to 1 ml serum-free Opti-MEM (Invitrogen/Gibco/life Technologies, Paisly, UK). Supernatants were harvested after 48 h and 50 µl of each sample was applied to an antibody-coated A2M ELISA plate (Alpha 2 Macroglobulin Human ELISA kit, Abcam) according to instructions of the manufacturer. Absorbance at OD_450_ was recorded.

### Proliferation assay and cell density assays

A dye dilution assay using the cell proliferation dye eFlour670 (eBioscience, CA, USA), equal to CFSE staining but GFP positive cells compatible, was used to test effects on proliferation. HUVEC were plated in 12 well plates and infected with Ad.con or Ad.MEF2C using a MOI of 10 or left uninfected. After 24 h cells were loaded with eFlour670 using a 2 µM solution in PBS containing Mg^2+^ and Ca^2+^ (Sigma-Aldrich) for 10 min at 37°C in the dark, subsequently washed 3x with full medium and cultured for 1 h (starting control) or for 48 h in proliferation medium (EGM2-MV or EBM+2% FCS+VEGF-A+bFGF (50 ng/ml each)). Then cells were trypsinized, washed in PBS with 10% FCS, fixed in 4% paraformaldehyde at room temperature for 15 min and analyzed in a BD FACScanto flow cytometer. Actively divided cells were determined by comparing 48 h and 1 h time points for dye dilution.

To score the increase in cell densities the SRB assay was used. HUVEC were infected with adenoviruses using a MOI of 10. After 24 h cells were trypsinized and seeded into 96-well plates (5000 cells/well) in quintuplicates for one, two or three days. Proliferation was determined by measuring the total protein content using the sulforhodamine B (SRB) colorimetric assay [18]. In short, cells were fixed with 50% trichloroacetic acid and stored at 4°C. Fixed cells were washed with H_2_O and stained with 0,4% SRB in 1% acetic acid at room temperature for 20 min. After a washing step with 1% acetic acid, the wells were air-dried and the SRB was solubilized in 10 mM Tris-Base solution. For quantification, absorbance was measured at 492 nm.

### Wound healing assay

HUVEC were infected with adenoviruses using a MOI of 10. Cells were trypsinized after 24 h, seeded into 24-well plates in triplicates and allowed to grow dense overnight. Then the cell monolayer was scratched with a yellow pipette tip forming a cross. Pictures were taken immediately after scratching and following cultivation of the cells for 24 h in full media with a Nikon Diaphot TMD microscope and a CCD camera (Kappa GmbH, Gleichen, Germany).

Percentage of refill of the wounded area was assessed using the Image J software (http://www.uhnres.utoronto.ca/facilities/wcif/imagej/).

### Spheroid-based sprouting assay using transduced cells

HUVEC were infected with adenoviruses using a MOI of 3 to 5 or transduced with lentivirus-containing supernatants at 1∶2 dilutions and further processed for the spheroid-based sprouting assay 4 h after infection with adenoviruses or 48 h after transduction with lentiviruses. In certain cases HUVEC were first transduced with lentiviruses for 48 h, then the cells were in addition infected with adenoviruses and 4 h later used for the spheroid sprouting assay. The spheroid based sprouting assay was performed as described in detail in ref. [19]. In short, cells were suspended in growth medium containing 0.25% (wt/vol) methylcellulose (Viscosity 4,000 cP, Sigma-Aldrich) and incubated in hanging drops overnight to obtain single spheroids of approximately 400 cells. These were embedded into a gel obtained by mixing equal volumes of a rat collagen solution and a methylcellulose solution followed by addition of FCS to a final concentration of 10%. A tenth of volume basal endothelial medium containing VEGF-A and bFGF (for final concentration 50 ng/ml each) or without growth factors was layered on top of the polymerized gel. After 24 h total sprout length was measured on pictures taken on the Nikon microscope with ImageJ software. For statistical quantification at least 15 spheroids per well were analyzed.

### Spheroid sprouting assay using conditioned cell culture supernatant

HUVEC were infected with adenoviruses using a MOI of 5 to 10 for 24 h, or transduced with lentivirus-containing 293T cell supernatants for 24 h and subsequently in addition infected with adenovirus for 24 h. Then medium was replaced by fresh serum-free Opti-MEM medium (Invitrogen) and the cells further incubated for 48 h. The conditioned cell supernatants were then collected and concentrated by centrifugation at 2500 rpm for 30 min in a benchtop cell culture centrifuge using a Vivaspin 20 spin-column with a 50 kDa cut-off membrane (Sartorius, Göttingen, Germany). Samples of the cell culture supernatant or of a A2M solution in PBS were added to the methocel fraction following addition of the spheroids before collagen gel formation. Sprouting was stimulated by overlaying the formed gel with VEGF-A and bFGF (final concentration 50 ng/ml each).

### Statistics

Results from single experiments performed in triplicates are described as mean ± standard deviation of the mean (SD). Combined results from several individual experiments including umbilical cord blood units from different donors are shown as mean ± standard error of the mean (SEM). Statistical analysis was performed using Student's t-test. A p-value of <0.05 was considered as statistically significant.

## Results

### The VEGF inducible transcription factor MEF2C inhibits angiogenic sprouting

**Figure 1:**
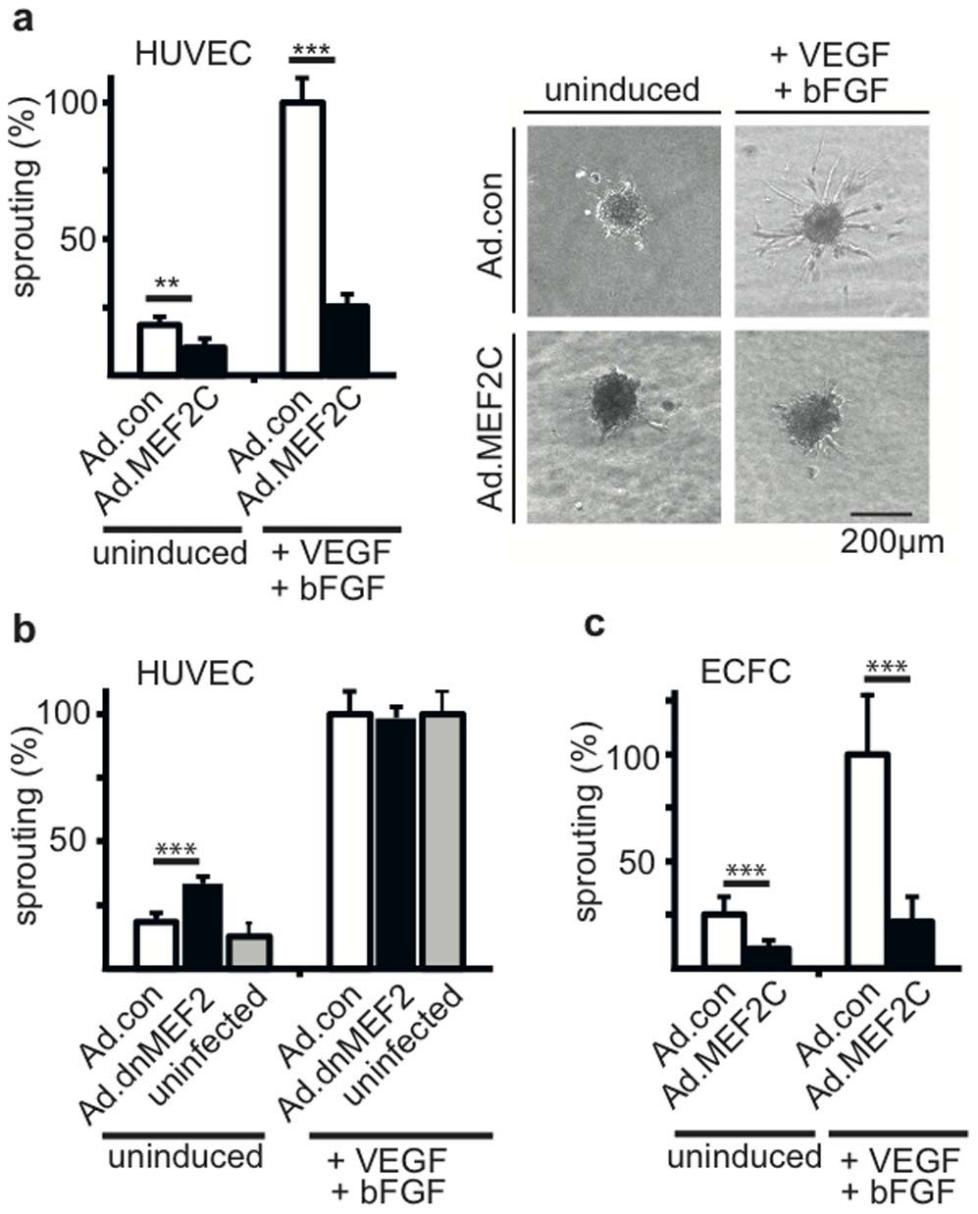
MEF2C negatively controls angiogenic sprouting. HUVEC or ECFC were infected with recombinant adenoviruses encoding MEF2C or a dominant negative version of MEF2C (Ad.MEF2C, Ad.dnMEF2C) or empty control viruses (Ad.con) as indicated using MOIs of 3 to 10. Cell spheroids were generated, embedded into collagen gels and induced with VEGF and bFGF (50 ng/ml) or cultured without induction as described in the Methods section. Sprouts were allowed to form for 24 hours. Subsequently pictures were taken for analyses and total sprout length per spheroid assessed using ImageJ software. Data were calculated from a minimum of 15 spheroids per condition and displayed as mean values ± SEM. (a) depicts the effect of MEF2C on HUVEC as calculated from seven independent experiments. The left panel shows the statistical analysis, the right panel exemplary spheroids. (b) shows the effect of a dominant negative version of MEF2 on HUVEC and (c) displays the effect of MEF2C on ECFC as calculated from three independent experiments each. In all parts mean sprout formation induced by VEGF-A and bFGF in Ad.con infected cells is arbitrarily set to 100%. **p<0.005, ***p<0.001.

We have previously demonstrated, that the transcription factor MEF2C is specifically inducible by the pro-angiogenic mediators VEGF-A and, to some extent, also by bFGF, but not by other inflammatory or more broadly active growth stimuli such as IL1 or EGF, respectively, implying a function during angiogenesis [6], [20]. To delineate a potential function of MEF2C during angiogenesis, an in-gel spheroid sprouting assay was conducted with human umbilical vein endothelial cells (HUVEC) infected with an adenovirus driving overexpression of MEF2C. Intriguingly, MEF2C drastically inhibited VEGF and bFGF induced sprouting as well as basal sprout formation (Fig. 1a). This inhibition was also found when sprouting was solely induced by either VEGF-A or bFGF (Fig. S1), however the combination continuously evoked solid induction of sprouting in the assay. In line with an inhibitive effect of MEF2C, the overexpression of a dominant-negative MEF2 had a rather stimulating effect on basal sprouting of HUVEC (Fig. 1b). Infection with control adenoviruses did not significantly influence sprout formation compared to uninfected HUVEC (Fig. 1b).

A similar inhibitive effect of MEF2C on sprouting was observed when late endothelial progenitor cells (late blood outgrowth endothelial cells, BOEC, also termed endothelial colony forming cells, ECFC) [21] which characteristically display a higher sprouting potential than vessel wall-derived endothelial cells like HUVEC, were examined (Fig. 1c). This further emphasizes a repressive function of MEF2C for progenitor and mature endothelial cells.

**Figure 2:**
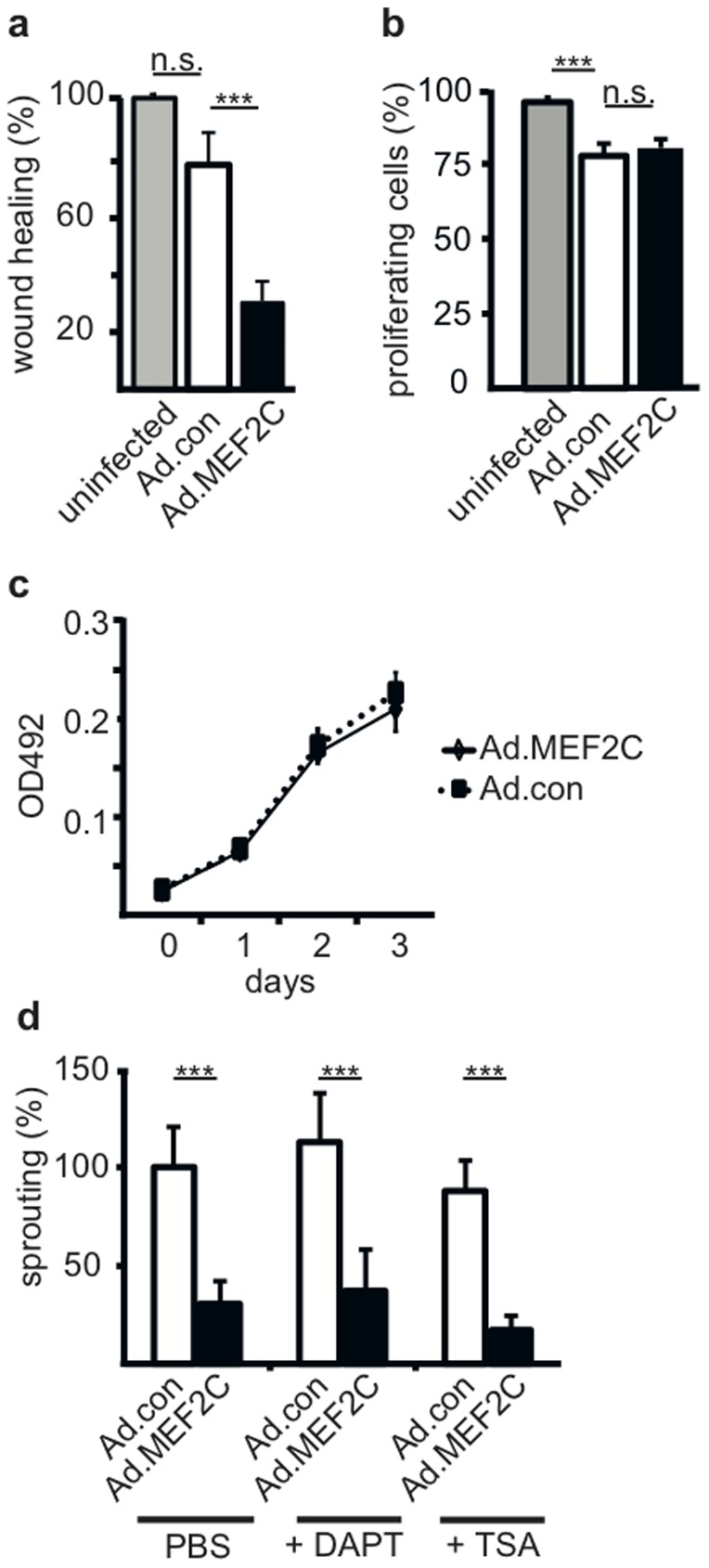
MEF2C primarily affects migration, but not proliferation, and the effect is independent from Notch signaling and histone acetylase activity. (a) Inhibition of migration in a wounding assay. A monolayer of HUVEC infected for 48 hours with Ad.MEF2C or Ad.con or left uninfected was scratched and the relative refill of the wounded area was scored and quantified using ImageJ software. The shown results were calculated from three independent experiments performed in triplicates and are displayed as mean values ± SEM. Values obtained from uninfected HUVEC were arbitrarily set to 100%. (b) Absence of a significant effect of MEF2C on actively proliferating cells. The percentage of proliferating cells was assessed for HUVEC seeded in 12 well culture plates and infected with Ad.con, Ad.MEF2C or left uninfected. 24 hours after infection cells were loaded with 2 µM cell proliferation Dye eFlour670, cultured for further 48 hours in complete growth medium and divided cells determined by flow cytometry. Data were calculated from three independent experiments performed in triplicates and displayed as mean values ± SD. (c) Absence of a substantial effect of MEF2C on the increase of cell density. To assess the increase in cell density over a several days period the SRB assay was performed as described in the Methods section. Total protein content was measured after 0, 1, 2 and 3 days of culturing HUVEC infected with Ad.con or Ad.MEF2C in full growth medium in 96 well plates. Data were calculated from three independent experiments performed in quintuplicates and displayed as mean values ± SEM. (d) Effects of inhibition of Notch signaling by DAPT and of histone acetylase activity by TSA. HUVEC were infected with Ad.MEF2C or Ad.con using a MOI of 10. Then they were used in the spheroid sprouting assay stimulated by VEGF and bFGF (50 ng/ml each). Where indicated DAPT (40 µM) or TSA (100 ng/ml) was added. Shown data are mean values ± SD and were calculated from one representative experiment out of three independent experiments performed. Ad.con infected HUVEC induced with VEGF and bFGF but without DAPT or TSA addition were arbitrarily set to 100%. ***p<0.001.

Sprout formation is a multifunctional process, which involves migration as well as proliferation of endothelial cells. We therefore elucidated the influence of MEF2C on migratory capacity of HUVEC in a scratch wound healing assay. Overexpression of MEF2C reduced the potential of endothelial cells to refill a cell-deprived area of a monolayer indicating impaired migration (Fig. 2a). However, elevated presence of MEF2C in HUVEC did not alter cell division rates assessed in a dye dilution assay conducted in complete endothelial growth medium (Fig. 2b). Similarly, when cells were induced solely by VEGF-A/bFGF and the analysis was restricted to infected (GFP-gated) cells no effect of MEF2C was detectable (Fig. S2). Furthermore, the capacity for robust growth to density was determined in a SRB assay over three days, indicating no decrease of growth and viability by overexpression of MEF2C (Fig. 2c). This supports that MEF2C selectively inhibits migratory processes, but leaves proliferation unaffected.

Considering that on the one hand Notch signaling is important during angiogenesis [3] and MEF2C was reported to bind the Notch intracellular domain in cardiomyocytes [22], [23], we tested whether inhibition of Notch signaling by DAPT would alter the inhibitive effect of MEF2C on angiogenic sprouting. On the other hand, it has been reported that class II HDAC can mediate repression of MEF2C-dependent genes [24], Therefore, we also evaluated inhibition of HDAC activities by Trichostatin A (TSA). DAPT was applied in concentrations (40 µM) that activated basal sprouting of endothelial cells indicative of a blockage of inhibitive Notch signaling (Fig. S3a). TSA was used in concentrations (100 ng/ml) as described for inhibition of HDACs [25] that would not yet generally impair endothelial viability and sprouting as it became visible at higher concentrations (Fig. S3b). However, inhibition of gamma-secretase/Notch signaling or inhibition of HDAC activity could not change the inhibitive effect of MEF2C on angiogenic sprouting (Fig. 2d) suggesting that the observed MEF2C effects do neither involve Notch signaling nor HDAC dependent regulation.

### MEF2C upregulates alpha-2-macroglobulin (A2M) production by endothelial cells

**Figure 3:**
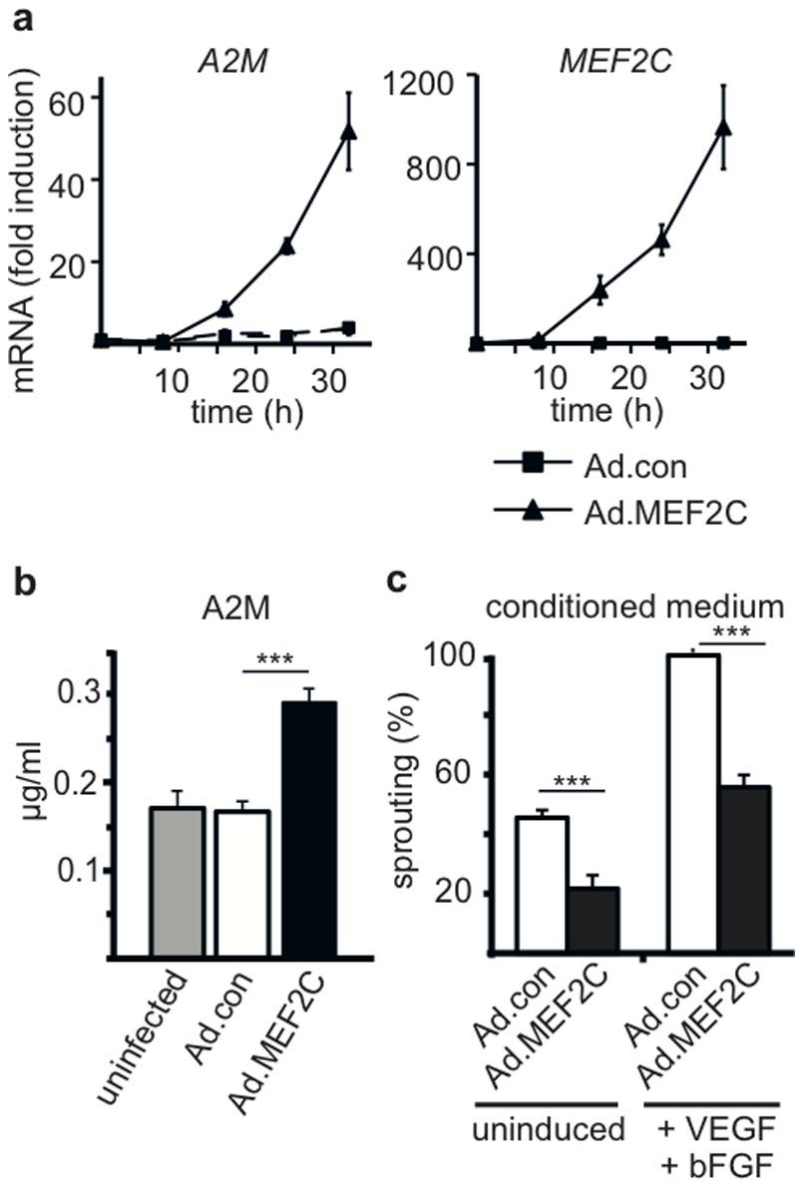
Alpha-2-macroglobulin (A2M) is upregulated by MEF2C in HUVEC on mRNA and protein level. (a) *A2M* and *MEF2C* mRNA levels. HUVEC were transduced for 8, 16, 24 and 32 hours with Ad.MEF2C or Ad.con using MOI of 10 or left without infection (0 hours value). RNAs were isolated from the cells and *A2M* as well as *MEF2C* mRNA levels were determined by realtime RT-PCR as described in detail in the Methods section. Fold induction levels are shown as mean values ± SD calculated from triplicates of one representative experiment out of three independent experiments performed. Obtained values were normalized to beta-2-microglobulin mRNA as internal standard. (b) A2M protein. HUVEC were infected with Ad.MEF2C, Ad.con or left without infection for 8 hours, then medium was changed to serum-free Opti-MEM medium and supernatants were harvested after 48 hours. A2M secreted into supernatants was determined using a commercial A2M ELISA kit and a standard curve obtained with purified A2M. The mean concentration of A2M ± SD as calculated from three HUVEC batches analyzed is shown. (c) Supernatants of MEF2C virus transduced HUVEC inhibit sprouting. HUVEC were transduced with Ad.con or Ad.MEF2C or left uninduced for 24 hours, then medium was changed to Opti-mem, after 48 hours supernatants were collected and further concentrated by diafiltration as described in the Methods section. These conditioned media were added to the sprouting assay (50–70 µl to a 500 µl assay) and VEGF-A/bFGF-induced as well as basal sprout formation was scored . Data are displayed as mean values ± SEM as calculated from 3 independent experiments. The values obtained for conditioned medium from Ad.con infected cells treated with VEGF and bFGF were arbitrarily set to 100%. ***p<0.001.

As a transcription factor MEF2C was expected to convey its effects via regulation of gene expression. We have therefore delineated by gene expression profiling which genes are induced following adenoviral overexpression of MEF2C in HUVEC. This revealed that *A2M* is by far the most highly induced gene in HUVEC infected with Ad.MEF2C for 32 hours. Gene upregulation by MEF2C was very selective and comprised only a small number of genes with induction rates over 3-fold (Table S1). The complete set of data is submitted to GEO under accession number GSE46279. The strong upregulation of *A2M* mRNA by MEF2C was confirmed by repeated realtime RT-PCR analyses displaying a 60-fold increase 30 hours after transduction with Ad.MEF2C (Fig. 3a). Furthermore, when we tested A2M protein by ELISA in cell culture supernatants a significant increase in A2M levels was observed 56 hours after transduction with Ad.MEF2C. This supports that the strongly increased levels of *A2M* mRNA are translated and lead to an increase in A2M secretion (Fig. 3b).

### A2M represses angiogenic sprouting

We next evaluated whether supernatants from cultures transduced with Ad.MEF2C would inhibit sprouting when added to the spheroid sprouting assay. Indeed, supernatants of these cells reduced sprouting activity by half when compared to supernatants of control virus transduced cultures (Fig. 3c). In control experiments we confirmed that in these supernatants no significant amounts of viral particles were remaining that could lead to upregulation of MEF2C and mediate this suppression (Fig. S4). The inhibition should thus be mediated by soluble mediators secreted from MEF2C overexpressing cells.

**Figure 4:**
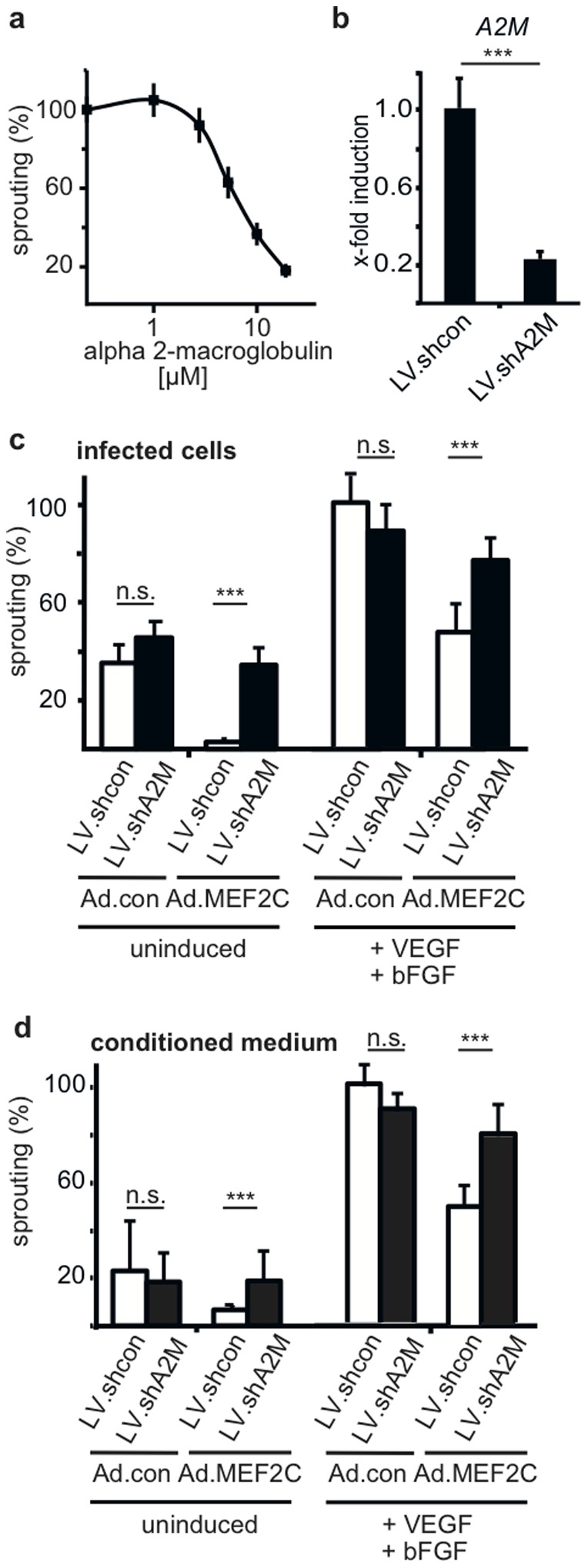
A2M mediates the inhibitive effect of MEF2C on sprouting. (a) Inhibition of sprouting by purified A2M. Increasing concentrations of commercially available A2M were added to the spheroid sprouting assay and the effects on total sprout length scored. Data are displayed as mean values ± SEM as calculated from three experiments. (b) A2M mRNA expression is strongly reduced upon shRNA mediated knockdown. HUVEC were transduced with LV.shA2M or LV.shcon for 48 hours. Total RNA was isolated, subjected to cDNA synthesis and analyzed by realtime RT-PCR. Values were normalized to beta-2-microglobulin mRNA as internal standard and displayed as mean ±SD. One representative experiment of 3 performed in triplicates is shown (c) Knockdown of A2M restores sprouting activity in Ad.MEF2C transduced HUVEC. Cells were first transduced with LV.shcon or LV.shA2M for 24 hours and then infected with Ad.con or Ad.MEF2C for 6 hours before they were used to form spheroids for the spheroid sprouting assay. Basal and VEGF-A/bFGF-induced sprouting was scored. Data displayed are mean values ± SEM calculated from three experiments. (d) Knockdown of A2M reduces the inhibitory activity in conditioned media from Ad.MEF2C transduced cells. HUVEC were transduced with LV.shcon or LV.shA2M for 24 hours and then infected with Ad.con or Ad.MEF2C for 8 hours. Then medium was changed to serum-free Opti-MEM medium, supernatants were harvested after 48 hours, concentrated by diafiltration and added to the spheroid sprouting assay. Basal and VEGF-A/bFGF-induced sprouting was assessed. Values depicted are mean values ± SEM calculated from three independent experiments. The values obtained for conditioned medium from Ad.con infected cells treated with VEGF and bFGF were arbitrarily set to 100%. n.s. not significant, ***p<0.001.

When we tested commercially available A2M protein purified from human serum a strong reduction of sprouting was observed confirming that A2M is capable to inhibit sprouting in a concentration-dependent manner (Fig. 4a). This is in line with reports that A2M can bind and sequester VEGF and bFGF and is further capable to neutralize serine proteases such as metalloproteinases involved in the invasion process during angiogenic sprouting [26]–[29].

In order to confirm that A2M is indeed the mediator of the inhibitive effect of MEF2C we tested knock-down of A2M employing an A2M shRNA strategy (Fig. 4b). HUVEC were first transduced with shRNA expressing lentiviruses, then infected with Ad.MEF2C or Ad.con and finally subjected to the spheroid sprouting assay. Again, MEF2C overexpression in the presence of control shRNA strongly reduced basal as well as VEGF-A/bFGF induced sprouting. However, when A2M induction by MEF2C was prevented by A2M shRNA-mediated knock-down, sprouting activity was largely re-established (Fig. 4c). In an analogous setting we further evaluated whether this effect would also become apparent when we would measure the inhibitive potential of the supernatants. Again, cells were transduced with the shRNA lentiviruses followed by the overexpression adenoviruses and cell culture supernatants were collected. When we tested the capacity of these supernatants to inhibit sprouting in the spheroid sprouting assay, a similar result as described before was obtained. Conditioned medium from cultures overexpressing MEF2C in the presence of control shRNA displayed the usual about 50% inhibition. However, when A2M expression was diminished with *A2M* shRNA, control sprouting activity was also nearly restored (Fig. 4d).

Taken together, these results clearly demonstrate that upregulation of A2M mediates, at least to a large extent, the inhibition of angiogenic sprouting of endothelial cells caused by MEF2C.

### Induction of MEF2C and A2M is reduced under hypoxic conditions which facilitates increased sprouting

**Figure 5:**
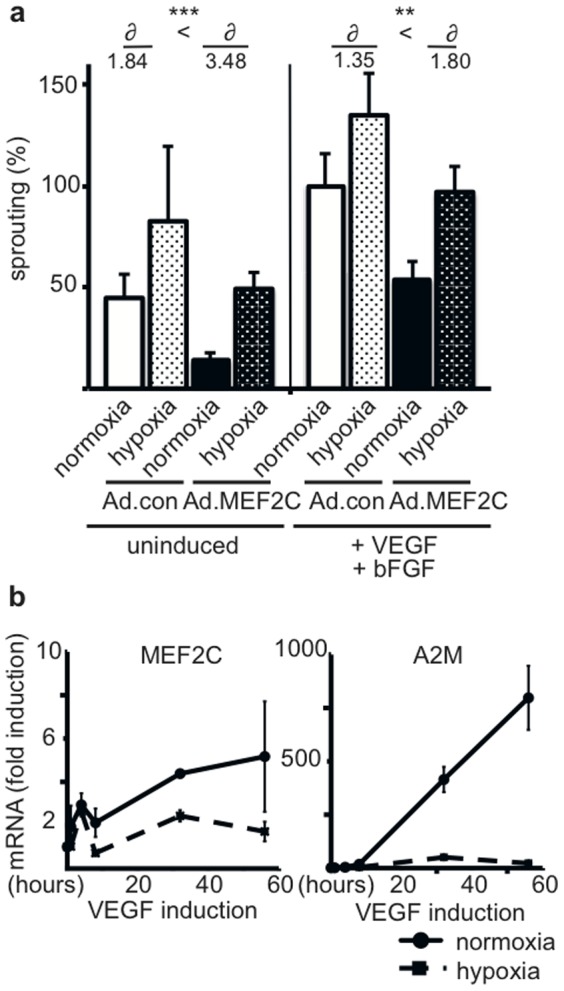
Under hypoxia the inhibition of sprouting by MEF2C is relieved and VEGF-dependent induction of *A2M* mRNA is abolished. (a) Reduction in MEF2C-mediated inhibition of sprouting under hypoxia. HUVEC were transduced with Ad.MEF2C or control adenoviruses and incubated for 48 hours. Then the spheroid sprouting assay was performed without or with addition of VEGF and bFGF as inducers under normoxia or at 1.5% oxygen in the cell culture incubator. 15 spheroids were analyzed per condition. Shown data were calculated from 4 independent experiments and displayed as mean values ± SEM. The values obtained for Ad.con infected cells treated with VEGF and bFGF under normoxia were arbitrarily set to 100%. ∂ indicates difference between hypoxia and normoxia, which is significantly stronger pronounced in Ad.MEF2C transduced HUVEC. **p<0.005, ***p<0.001 (b) Reduction in VEGF-mediated upregulation of *MEF2C* and *A2M* mRNA under hypoxia. HUVEC were stimulated with VEGF-A and further incubated under either normoxic or hypoxic conditions for 4, 8, 32 and 56 hours. Total RNA was isolated, subjected to cDNA synthesis and analyzed by realtime RT-PCR. Values were normalized to beta-2-microglobulin mRNA as internal standard and displayed as mean ±SD. One representative experiment of 3 performed in triplicates is shown.

Sprouting angiogenesis normally occurs in hypoxic tissues [30]. We therefore evaluated whether MEF2C-mediated inhibition of sprouting would also be detectable under hypoxic conditions. The spheroid sprouting assay was performed with HUVEC overexpressing MEF2C under hypoxic conditions (1.5% oxygen). As expected hypoxia by itself led to an increase in basal and in VEGF-A/bFGF-induced sprouting. Importantly, the MEF2C-mediated inhibition of basal as well as VEGF-A/bFGF-induced sprouting was relieved under hypoxic conditions and sprouting activity was partly restored. The difference of induction from normoxia to hypoxia was much higher in the MEF2C overexpressing than in the control HUVEC. Hypoxia therefore largely rescued the inhibitive effect of MEF2C expression (Fig. 5a).

We then evaluated whether the rescue from inhibition would be due to reduced induction of MEF2C and A2M by VEGF. Indeed, HUVEC cultured under hypoxic conditions revealed a strongly reduced VEGF-A-mediated inducibility of *MEF2C* and *A2M* upregulation under hypoxia was largely abrogated (Fig. 5b). This strongly suggests that VEGF-A/bFGF upregulates the MEF2C/A2M axis mainly during normoxic conditions, however when hypoxia is established this negative feed-back loop is inactivated.

## Discussion

The MADS box containing transcription factor MEF2C has been shown to be involved in many different developmental processes, an important example is its role in cardiac muscle differentiation and cardiogenesis [31]–[33]. Our previous data have displayed that MEF2C is specifically induced in endothelial cells by angiogenic growth factors, primarily VEGF-A, implying a role of MEF2C in the control of angiogenesis [6]. Surprisingly, gain-of-function experiments of MEF2C revealed not a triggering, but a strong inhibitive effect on angiogenic sprout formation of endothelial cells (Fig. 1). This inhibitive effect was observed for mature endothelial cells derived from the vessel wall (HUVEC), as well as for late stage endothelial progenitor cells (BOEC/ECFC), implying that MEF2C serves a negative feed-back inhibitory function in progenitor and mature endothelial cells. When we tested migratory processes versus proliferation only migration, but not proliferation of the cells was affected. This suggests that rather the migration of tip cells than the proliferation of stalk cells would be affected in the growing sprout (Fig. 2 a–c).

Next we have been interested to identify the target genes of MEF2C, which would mediate the inhibitive effect. Gene expression profiling of cells overexpressing MEF2C revealed a limited, but specific response, less than 10 genes were upregulated by MEF2C more than 3-fold (Table S1). This is in contrast to our experiences using overexpression of other transcription factors, which frequently lead to a much broader effect [10], [34]. This could be interpreted that MEF2C either has only a small restricted number of specific target genes in endothelial cells or that a cofactor for MEF2C-directed transcription is limiting.

Alternatively, MEF2C function on certain genes could be modulated by proteins such as class II HDACs, which are known to mediate repression of MEF2-dependent genes [24]. However, relieve from potential HDAC-mediated inhibition by Trichostatin A did not alter the inhibitive functions of MEF2C on sprouting (Fig. 2d), suggesting that HDAC-mediated repression is not the cause for the inhibitory effects of MEF2C. Vice versa, it has been shown by Urbich et al. [35] that the inhibition of angiogenic processes exerted by for example HDAC5 is not dependent on binding to MEF2C, supporting independent pathways for HDAC- and MEF2C-mediated inhibition.

We therefore assumed that rather the direct upregulation of a gene would cause the observed MEF2C effect. In this regard, one gene, *A2M*, stood out by its over tenfold upregulation by MEF2C in the gene profiling experiment and so being by far the most strongly induced (Table S1). It encodes alpha-2-macroglobulin (A2M), known as a serum protein, which forms homotetramers of about 700 kDa and is constitutively produced in the liver. However, its production by endothelial cells has to our knowledge so far not been described. A2M is known as a global serine protease inhibitor as well as a carrier protein for different growth factors, among them VEGF-A and bFGF [26]–[29]. Since angiogenesis is characterized by synthesis of matrix metalloproteases (MMPs) functioning in the degradation of the extracellular matrix (ECM) and allowing the endothelial sprout to invade the surrounding tissue, it seemed conceivable that A2M mediates the inhibitive effects of MEF2C. A2M could function, when secreted basolateral and luminal from the endothelial cells, by inhibiting protease activity and possibly also by sequestering proangiogenic factors from the binding to receptors on the endothelial cells. Further work was therefore focused on the potential role of A2M.

First we were able to show, by realtime RT-PCR analysis and ELISA assays, that endothelial cells upon MEF2C expression were indeed increasing the production of *A2M* mRNA about 50-fold and also secreted significant amounts of A2M protein (Fig. 3a,b). It should be noted that also without MEF2C overexpression endothelial cells contained detectable amounts of *A2M* mRNA indicating that lower amounts of A2M are constantly produced by cultured endothelial cells in the quiescent state.

When A2M was mediating the effect of MEF2C, supernatants obtained from cultures transduced with Ad.MEF2C and containing increased A2M levels should be able to inhibit sprouting. This was indeed the case (Fig. 3c). Furthermore, we showed that commercially available A2M purified from serum, when added to the sprouting assay, inhibited sprouting in a dose-dependent manner (Fig. 4a).

To directly confirm that MEF2C-induced A2M causes at least a substantial part of the observed suppression of sprouting a lentiviral knockdown strategy was employed that specifically depletes *A2M* mRNA accumulation after MEF2C overexpression. Indeed, when *A2M* mRNA production was diminished (Fig. 4b), the inhibitory effect of MEF2C was largely abrogated. This became apparent when transduced cells themselves were used in the spheroid assay as well as when only the supernatants of transduced cells were examined, thereby revealing a direct involvement of A2M in the observed suppressive role of MEF2C for sprouting angiogenesis (Fig. 4c,d). That this effect could be caused by potential re-infection with residual viral particles contained in the supernatants seems unlikely since no substantial increase of MEF2C mRNA was detectable in cells after addition of supernatants (Fig. S4). Furthermore, no GFP expression was detectable in the cells treated with supernatants.

In regard of A2M as a major mediator of MEF2C controlled inhibitory mechanisms, there are multiple reported evidences that A2M is involved in prevention of tissue damage. A correlation of pathologies like COPD (chronicle obstructive pulmonary disease), rheumatoid arthritis and even pancreatitis with A2M imbalances has been described which presumably results in excess of metalloprotease activities injuring tissue or allowing for metastatic tumor spreading [36]–[38].

A central mechanism to regulate sprouting via determining stalk versus tip cell phenotype is Notch signaling. Based on reports that MEF2C in muscle cells can interact with the Notch intracellular domain [22], [23], which functions as a transcription factor when cleaved from the transmembrane protein, it appeared possible that combinatorial MEF2C and Notch signaling could contribute to the inhibition of sprouting, e.g. by inducing genes favoring the non-sprouting stalk cell phenotype. However, abrogation of Notch signaling through the inhibitor DAPT did not alter the effects of MEF2C implicating that the MEF2C-mediated inhibition is independent of Notch signaling (Fig. 2d).

That MEF2 factors fulfill a role during angiogenic sprouting of endothelial cells also *in vivo*, is supported by several other independent recent findings. First, a MEF2C global knockout is embryonically lethal at day 10.5 due to cardiac as well as vascular defects [13]. Second, it has been demonstrated that MEF2C in cooperation with the transcription factor Klf2 can install cell quiescence and VEGF resistance, both potentially characteristics of the phalanx cells [39]. This was dependent on induction of Klf2 by MEF2C via the BMK1/ERK5 signaling pathway [40]. An interrelation between BMK1 signaling and MEF2C *in vivo* is further corroborated by the finding that an endothelial specific knock-out of BMK1 gives a most similar phenotype compared with the Mef2c global knock-out mice, i.e. both display embryonic lethality at day E10.5 and cardiac as well as vascular defects [41]. Third, Mef2c was shown to be overrepresented in tip cell enriched murine cornea tissue samples [12], but what kind of role Mef2c may play during tip-stalk cell selection was not further investigated in this study. Finally, further evidence for an endothelial role of MEF2C *in vivo* is provided by the use of recently bred mice with endothelial-specific Mef2c ablation in a model of oxygen-induced retinopathy. Ablation of Mef2c diminished oxygen-induced vessel loss and strongly promoted normal vascular regrowth reducing retinal avascularity. This is in line with an anti-angiogenic role of MEF2C under stress conditions *in vivo* [42].

In regard of cell-type specific knock-outs it was surprising that these mice with endothelial specific ablation of Mef2c were viable. It should however be noted, that this is a phenomenon observed for all tissue specific knock-outs of Mef2c tested so far, even for ablation in cardiomyocytes, for which ample other evidence for an essential role of MEF2C is available [43]. One possible explanation for this might be redundancy among the multiple MEF2 isoforms (A–D), which could potentially compensate detrimental effects.

It is a general principle that important biological processes are regulated by a balance of positive and negative mediators. Although on the first glance it appeared surprising that a gene upregulated by VEGF-A, the primary trigger of angiogenesis, was inhibiting angiogenesis, it seems plausible that the process of vessel formation is strictly controlled by negative regulators and these might be already co-induced by the major triggers of the process. In line with this possibility *MEF2C* mRNA is induced with somewhat delayed kinetics around 3 to 4 h after VEGF-A treatment when compared to mRNAs for transcription factors implicated in induction of angiogenesis such as EGR-3 and NR4A2 that show very rapid induction around the 0.5 to 1 h time point [6].

MEF2C is not the only factor induced preferentially by VEGF-A under normoxic conditions, as we have previously defined the homeobox factor HLX to similarly contribute to negative control of angiogenesis by upregulation of the negative guidance receptor UNC5B [10]. This underscores the importance of inhibitory mechanisms to keep neovessel formation under stringent control and is in line with the findings that excessive vascularization hallmarks many pathologies from chronic inflammatory diseases to cancer [44].

A most attractive hypothesis is that the MEF2C/A2M axis contributes to adapting the sprouting activity to the oxygen gradient. Hypoxia, the deficiency of oxygen is the initial trigger to produce VEGF-A and consecutively new blood vessels [30]. Endothelial cells are highly adapted to function under metabolic stress conditions such as hypoxia. However, the transcriptional response to VEGF-A is obviously very different under hypoxic conditions when compared to normoxia. In regard of MEF2C/A2M the induction of MEF2C is much less pronounced and A2M upregulation is completely prevented. This strongly suggests that the MEF2C/A2M pathway has a major role in preventing inappropriate sprouting under normoxic conditions and to shut down angiogenesis when appropriate oxygen supply is restored following neovascularization.

## Supporting Information

Figure S1 **MEF2C negatively controls angiogenic sprouting stimulated by VEGF, bFGF and a combination of VEGF and bFGF.** HUVEC were infected with Ad.MEF2C or Ad.con using MOIs of 3 to 10. Cell spheroids were generated, embedded into collagen gels and induced as described in the Methods section either with VEGF (50 ng/ml), bFGF (50 ng/ml), a combination of VEGF (50 ng/ml) and bFGF (50 ng/ml) or were cultured without induction. Sprouts were allowed to form for 24 hours. Subsequently pictures were taken for analyses and total sprout length per spheroid assessed using ImageJ software. Data were calculated from a minimum of 10 spheroids per condition and displayed as mean values ± SD. Sprout formation induced by VEGF and bFGF in Ad.con infected cells is arbitrarily set to 100%.
(TIFF)

Figure S2 **MEF2C does not affect proliferation triggered solely by VEGF-A and bFGF.** The percentage of proliferating cells was determined for HUVEC infected with Ad.con, Ad.MEF2C or left uninfected. Cells were loaded with cell proliferation dye eFlour670 24 h following infection. After further culturing in EBM containing 2% FCS, 50 ng/ml VEGF-A and 50 ng/ml bFGF for 48 h divided cells were scored by flow cytometry. Data were calculated from three independent experiments performed in triplicates and shown as mean values ±SD.
(TIFF)

Figure S3 **Applied concentrations of DAPT induce basal sprouting and concentrations of TSA were selected to allow sprouting without impairment of sprouting.** HUVEC were tested in the spheroid sprouting assay and stimulated by VEGF and bFGF (50 ng/ml) or left uninduced. (a) Inhibition of Notch signaling by DAPT induces basal sprouting. Where indicated DAPT (40 µM) was added. (b) Effects of TSA on sprouting of HUVECs. HUVEC were tested in the presence of TSA at 100 ng/ml or 500 ng/ml concentration. Shown data are mean values ± SD and were calculated from one representative experiment out of three independent experiments performed. HUVEC induced with VEGF and bFGF but without DAPT or TSA addition were arbitrarily set to 100%.
(TIFF)

Figure S4 **Concentrated supernatants derived from Ad.MEF2C infected cells do not contain residual adenovirus leading to overexpression of MEF2C mRNA.** HUVEC were infected with Ad.MEF2C, Ad.con or left without infection for 8 hours, then medium was changed to serum-free Opti-MEM medium. Supernatants were harvested after 48 hours and further concentrated by diafiltration as described in the Methods section. Concentrated supernatants were added to monolayers of HUVECs and the cells cultured for 24 h, an equal period as used for sprout formation. Then RNA was isolated and subjected to realtime RT-PCR analysis as described in the Method section. Relative mRNA levels are shown as mean values ± SD calculated from duplicates of three independent experiments. Obtained values were normalized to beta-2-microglobulin mRNA as internal standard.
(TIFF)

Table S1 **List of genes upregulated over three-fold by MEF2C.** HUVEC were transduced with adenoviruses encoding MEF2C (Ad.MEF2C) or control adenoviruses without cDNA inserts (Ad.con) or cultured without virus transduction. 8, 16 and 32 h after transduction total RNA was isolated and subjected to microarray analysis using Affymetrix Human Gene Level 1.0 ST Gene Chips as described in the Methods section. Values for non-infected HUVEC (uninf) represent random expression intensities as measured in the microarray analysis. Changes in gene expression intensities induced in Ad.con- and Ad.MEF2C -infected cultures relative to non-infected HUVEC are displayed in the consecutive columns.
(DOCX)

## References

[1] Dvorak HF, Brown LF, Detmar M, Dvorak AM (1995) Vascular permeability factor/vascular endothelial growth factor, microvascular hyperpermeability, and angiogenesis. Am J Pathol 146: 1029–1039. 7538264 PMC1869291

[2] Semenza GL (2003) Targeting HIF-1 for cancer therapy. Nat Rev Cancer 3: 721–732. 13130303 10.1038/nrc1187

[3] Phng LK, Gerhardt H (2009) Angiogenesis: a team effort coordinated by notch. Dev Cell 16: 196–208. 19217422 10.1016/j.devcel.2009.01.015

[4] Hofer E, Schweighofer B (2007) Signal transduction induced in endothelial cells by growth factor receptors involved in angiogenesis. Thromb Haemost 97: 355–363. 17334501 PMC2879321

[5] Koch S, Tugues S, Li X, Gualandi L, Claesson-Welsh L (2011) Signal transduction by vascular endothelial growth factor receptors. Biochem J 437: 169–183. 21711246 10.1042/BJ20110301

[6] Schweighofer B, Testori J, Sturtzel C, Sattler S, Mayer H, et al (2009) The VEGF-induced transcriptional response comprises gene clusters at the crossroad of angiogenesis and inflammation. Thromb Haemost 102: 544–554. 19718476 10.1160/TH08-12-0830 PMC2886966

[7] Liu D, Jia H, Holmes DI, Stannard A, Zachary I (2003) Vascular endothelial growth factor-regulated gene expression in endothelial cells: KDR-mediated induction of Egr3 and the related nuclear receptors Nur77, Nurr1, and Nor1. Arterioscler Thromb Vasc Biol 23: 2002–2007. 14525795 10.1161/01.ATV.0000098644.03153.6F

[8] Zhao D, Desai S, Zeng H (2011) VEGF stimulates PKD-mediated CREB-dependent orphan nuclear receptor Nurr1 expression: role in VEGF-induced angiogenesis. Int J Cancer 128: 2602–2612. 20715116 10.1002/ijc.25600

[9] Liu D, Evans I, Britton G, Zachary I (2008) The zinc-finger transcription factor, early growth response 3, mediates VEGF-induced angiogenesis. Oncogene 27: 2989–2998. 18059339 10.1038/sj.onc.1210959

[10] Testori J, Schweighofer B, Helfrich I, Sturtzel C, Lipnik K, et al (2011) The VEGF-regulated transcription factor HLX controls the expression of guidance cues and negatively regulates sprouting of endothelial cells. Blood 117: 2735–2744. 21224470 10.1182/blood-2010-07-293209 PMC3096761

[11] Rivera CG, Mellberg S, Claesson-Welsh L, Bader JS, Popel AS (2011) Analysis of VEGF—a regulated gene expression in endothelial cells to identify genes linked to angiogenesis. PLoS One 6: e24887. 21931866 10.1371/journal.pone.0024887 PMC3172305

[12] del Toro R, Prahst C, Mathivet T, Siegfried G, Kaminker JS, et al (2010) Identification and functional analysis of endothelial tip cell-enriched genes. Blood 116: 4025–4033. 20705756 10.1182/blood-2010-02-270819 PMC4314527

[13] Bi W, Drake CJ, Schwarz JJ (1999) The transcription factor MEF2C-null mouse exhibits complex vascular malformations and reduced cardiac expression of angiopoietin 1 and VEGF. Dev Biol 211: 255–267. 10395786 10.1006/dbio.1999.9307

[14] Wojta J, Hoover RL, Daniel TO (1989) Vascular origin determines plasminogen activator expression in human endothelial cells. Renal endothelial cells produce large amounts of single chain urokinase type plasminogen activator. J Biol Chem 264: 2846–2852. 2492525

[15] Ingram DA, Mead LE, Tanaka H, Meade V, Fenoglio A, et al (2004) Identification of a novel hierarchy of endothelial progenitor cells using human peripheral and umbilical cord blood. Blood 104: 2752–2760. 15226175 10.1182/blood-2004-04-1396

[16] Green MR, Sambrook J (2012) Molecular cloning: a laboratory manual. Cold Spring Harbor, N.Y.: Cold Spring Harbor Laboratory Press. 3 v. (xxxiii, 1890, 1846 p.) p.1161, Calcium phosphate mediated Transfection of Cells with High-Molecular-Weight Genomic DNA.

[17] Tauber S, Jais A, Jeitler M, Haider S, Husa J, et al (2010) Transcriptome analysis of human cancer reveals a functional role of heme oxygenase-1 in tumor cell adhesion. Mol Cancer 9: 200. 20667089 10.1186/1476-4598-9-200 PMC2917430

[18] Vichai V, Kirtikara K (2006) Sulforhodamine B colorimetric assay for cytotoxicity screening. Nat Protoc 1: 1112–1116. 17406391 10.1038/nprot.2006.179

[19] Korff T, Augustin HG (1998) Integration of endothelial cells in multicellular spheroids prevents apoptosis and induces differentiation. J Cell Biol 143: 1341–1352. 9832561 10.1083/jcb.143.5.1341 PMC2133072

[20] Maiti D, Xu Z, Duh EJ (2008) Vascular endothelial growth factor induces MEF2C and MEF2-dependent activity in endothelial cells. Invest Ophthalmol Vis Sci 49: 3640–3648. 18450586 10.1167/iovs.08-1760 PMC4519038

[21] Critser PJ, Voytik-Harbin SL, Yoder MC (2011) Isolating and defining cells to engineer human blood vessels. Cell Prolif 44 Suppl 1: 15–21. 21481038 10.1111/j.1365-2184.2010.00719.x PMC3387928

[22] Pallavi SK, Ho DM, Hicks C, Miele L, Artavanis-Tsakonas S (2012) Notch and Mef2 synergize to promote proliferation and metastasis through JNK signal activation in Drosophila. EMBO J 31: 2895–2907. 22580825 10.1038/emboj.2012.129 PMC3395089

[23] Wilson-Rawls J, Molkentin JD, Black BL, Olson EN (1999) Activated notch inhibits myogenic activity of the MADS-Box transcription factor myocyte enhancer factor 2C. Mol Cell Biol 19: 2853–2862. 10082551 10.1128/mcb.19.4.2853 PMC84078

[24] Zhang CL, McKinsey TA, Chang S, Antos CL, Hill JA, et al (2002) Class II histone deacetylases act as signal-responsive repressors of cardiac hypertrophy. Cell 110: 479–488. 12202037 10.1016/s0092-8674(02)00861-9 PMC4459650

[25] Minucci S, Horn V, Bhattacharyya N, Russanova V, Ogryzko VV, et al (1997) A histone deacetylase inhibitor potentiates retinoid receptor action in embryonal carcinoma cells. Proc Natl Acad Sci U S A 94: 11295–11300. 9326603 10.1073/pnas.94.21.11295 PMC23446

[26] Mathew S, Arandjelovic S, Beyer WF, Gonias SL, Pizzo SV (2003) Characterization of the interaction between alpha2-macroglobulin and fibroblast growth factor-2: the role of hydrophobic interactions. Biochem J 374: 123–129. 12755687 10.1042/BJ20021655 PMC1223577

[27] Mehl JW, O'Connell W, Degroot J (1964) Macroglobulin from Human Plasma Which Forms an Enzymatically Active Compound with Trypsin. Science 145: 821–822. 14163324 10.1126/science.145.3634.821

[28] Bhattacharjee G, Asplin IR, Wu SM, Gawdi G, Pizzo SV (2000) The conformation-dependent interaction of alpha 2-macroglobulin with vascular endothelial growth factor. A novel mechanism of alpha 2-macroglobulin/growth factor binding. J Biol Chem 275: 26806–26811. 10862607 10.1074/jbc.M000156200

[29] Fontana V, Silva PS, Belo VA, Antonio RC, Ceron CS, et al (2011) Consistent alterations of circulating matrix metalloproteinases levels in untreated hypertensives and in spontaneously hypertensive rats: a relevant pharmacological target. Basic Clin Pharmacol Toxicol 109: 130–137. 21401887 10.1111/j.1742-7843.2011.00698.x

[30] Hickey MM, Simon MC (2006) Regulation of angiogenesis by hypoxia and hypoxia-inducible factors. Curr Top Dev Biol 76: 217–257. 17118268 10.1016/S0070-2153(06)76007-0

[31] Barbosa AC, Kim MS, Ertunc M, Adachi M, Nelson ED, et al (2008) MEF2C, a transcription factor that facilitates learning and memory by negative regulation of synapse numbers and function. Proc Natl Acad Sci U S A 105: 9391–9396. 18599438 10.1073/pnas.0802679105 PMC2453723

[32] Cante-Barrett K, Pieters R, Meijerink JP (2013) Myocyte enhancer factor 2C in hematopoiesis and leukemia. Oncogene. 10.1038/onc.2013.56 23435431

[33] Lin Q, Schwarz J, Bucana C, Olson EN (1997) Control of mouse cardiac morphogenesis and myogenesis by transcription factor MEF2C. Science 276: 1404–1407. 9162005 10.1126/science.276.5317.1404 PMC4437729

[34] Lucerna M, Pomyje J, Mechtcheriakova D, Kadl A, Gruber F, et al (2006) Sustained expression of early growth response protein-1 blocks angiogenesis and tumor growth. Cancer Res 66: 6708–6713. 16818645 10.1158/0008-5472.CAN-05-2732 PMC2882226

[35] Urbich C, Rossig L, Kaluza D, Potente M, Boeckel JN, et al (2009) HDAC5 is a repressor of angiogenesis and determines the angiogenic gene expression pattern of endothelial cells. Blood 113: 5669–5679. 19351956 10.1182/blood-2009-01-196485

[36] Mocchegiani E, Giacconi R, Costarelli L (2011) Metalloproteases/anti-metalloproteases imbalance in chronic obstructive pulmonary disease: genetic factors and treatment implications. Curr Opin Pulm Med 17 Suppl 1: S11–19. 22209925 10.1097/01.mcp.0000410743.98087.12

[37] Tchetverikov I, Ronday HK, Van El B, Kiers GH, Verzijl N, et al (2004) MMP profile in paired serum and synovial fluid samples of patients with rheumatoid arthritis. Ann Rheum Dis 63: 881–883. 15194590 10.1136/ard.2003.013243 PMC1755080

[38] Bisaro de Lorenc L, Ramos AM, Sanchez MC, Montenegro R, Chiabrando GA (2005) Structural evaluation of plasma alpha2-macroglobulin in acute pancreatitis. Clin Chem Lab Med 43: 1183–1189. 16232083 10.1515/CCLM.2005.205

[39] Wang W, Ha CH, Jhun BS, Wong C, Jain MK, et al (2010) Fluid shear stress stimulates phosphorylation-dependent nuclear export of HDAC5 and mediates expression of KLF2 and eNOS. Blood 115: 2971–2979. 20042720 10.1182/blood-2009-05-224824 PMC2854437

[40] Young A, Wu W, Sun W, Benjamin Larman H, Wang N, et al (2009) Flow activation of AMP-activated protein kinase in vascular endothelium leads to Kruppel-like factor 2 expression. Arterioscler Thromb Vasc Biol 29: 1902–1908. 19696400 10.1161/ATVBAHA.109.193540 PMC2766008

[41] Hayashi M, Kim SW, Imanaka-Yoshida K, Yoshida T, Abel ED, et al (2004) Targeted deletion of BMK1/ERK5 in adult mice perturbs vascular integrity and leads to endothelial failure. J Clin Invest 113: 1138–1148. 15085193 10.1172/JCI19890 PMC385403

[42] Xu Z, Gong J, Maiti D, Vong L, Wu L, et al (2012) MEF2C ablation in endothelial cells reduces retinal vessel loss and suppresses pathologic retinal neovascularization in oxygen-induced retinopathy. Am J Pathol 180: 2548–2560. 22521302 10.1016/j.ajpath.2012.02.021 PMC3378855

[43] Vong LH, Ragusa MJ, Schwarz JJ (2005) Generation of conditional Mef2cloxP/loxP mice for temporal- and tissue-specific analyses. Genesis 43: 43–48. 16106363 10.1002/gene.20152

[44] Carmeliet P (2005) Angiogenesis in life, disease and medicine. Nature 438: 932–936. 16355210 10.1038/nature04478

