# Supplementary figures and images for: The Transcription Factor MEF2C Negatively Controls Angiogenic Sprouting of Endothelial Cells Depending on Oxygen
Source: PLoS One. 2014 Jul 2;9(7):e101521. doi: 10.1371/journal.pone.0101521 (PMC4079651; doi:10.1371/journal.pone.0101521)

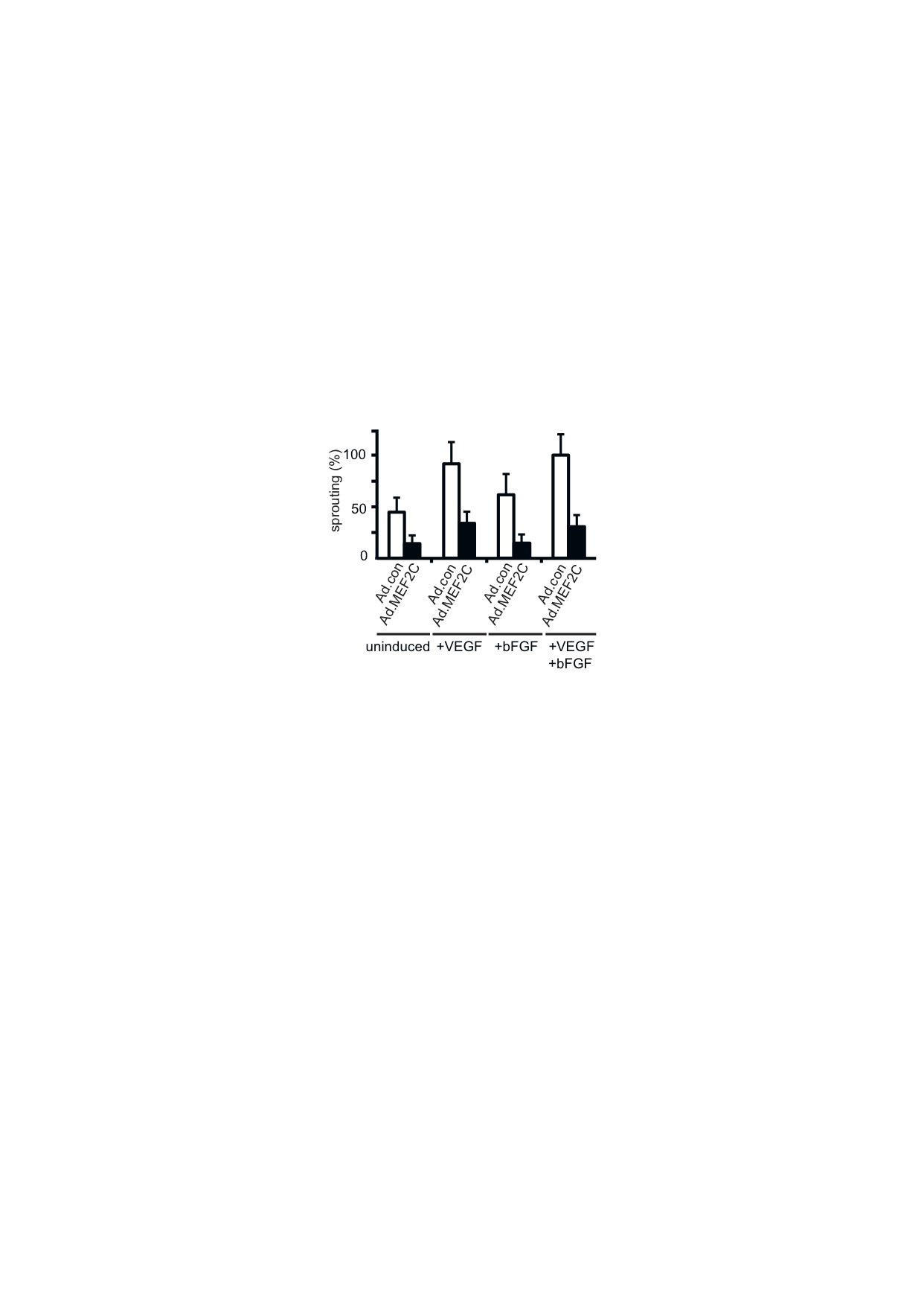

Supplement: Figure S1 — MEF2C negatively controls angiogenic sprouting stimulated by VEGF, bFGF and a combination of VEGF and bFGF. HUVEC were infected with Ad.MEF2C or Ad.con using MOIs of 3 to 10. Cell spheroids were generated, embedded into collagen gels and induced as described in the Methods section either with VEGF (50 ng/ml), bFGF (50 ng/ml), a combination of VEGF (50 ng/ml) and bFGF (50 ng/ml) or were cultured without induction. Sprouts were allowed to form for 24 hours. Subsequently pictures were taken for analyses and total sprout length per spheroid assessed using ImageJ software. Data were calculated from a minimum of 10 spheroids per condition and displayed as mean values ± SD. Sprout formation induced by VEGF and bFGF in Ad.con infected cells is arbitrarily set to 100%. (TIFF) [file pone.0101521.s001.tiff]

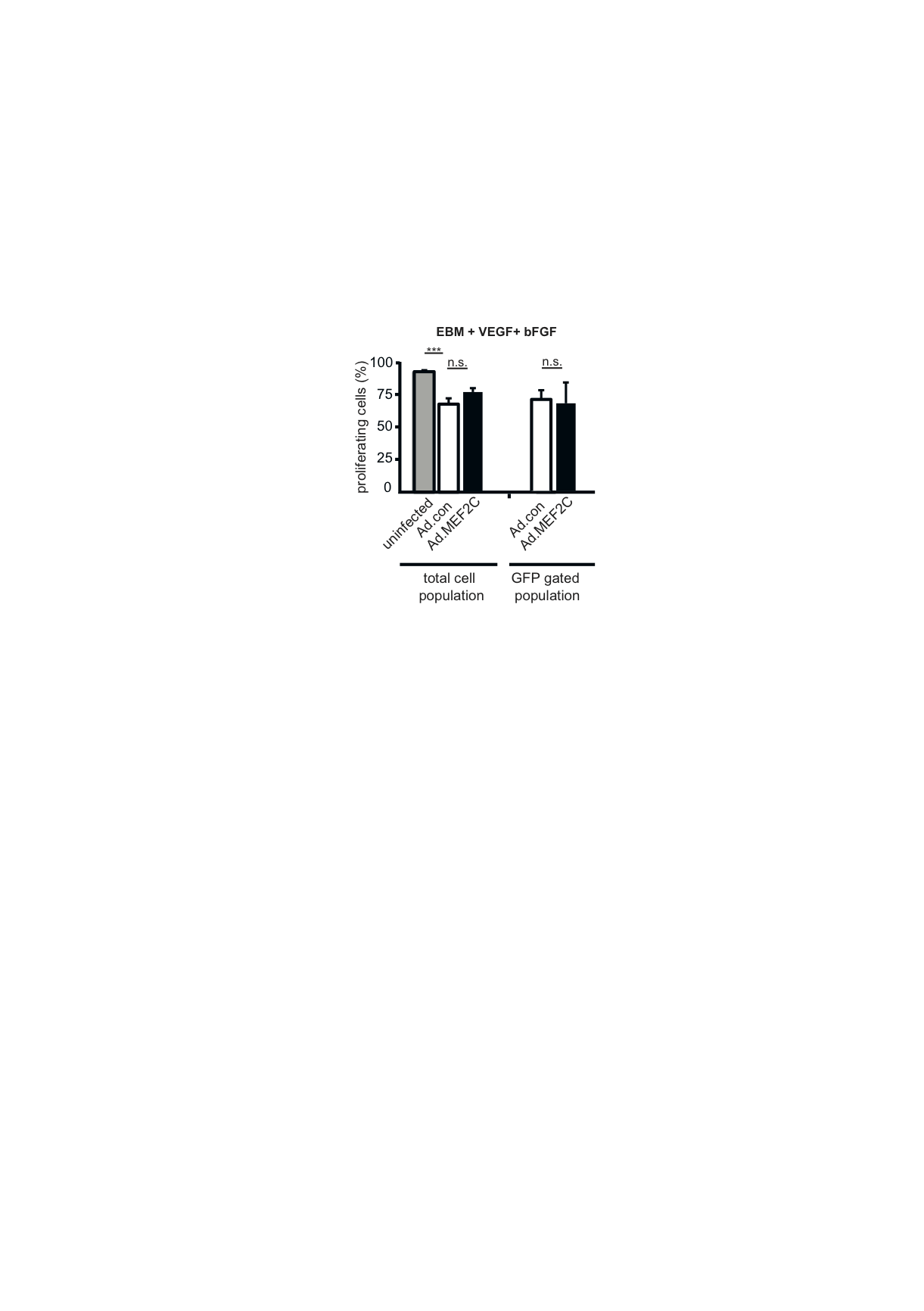

Supplement: Figure S2 — MEF2C does not affect proliferation triggered solely by VEGF-A and bFGF. The percentage of proliferating cells was determined for HUVEC infected with Ad.con, Ad.MEF2C or left uninfected. Cells were loaded with cell proliferation dye eFlour670 24 h following infection. After further culturing in EBM containing 2% FCS, 50 ng/ml VEGF-A and 50 ng/ml bFGF for 48 h divided cells were scored by flow cytometry. Data were calculated from three independent experiments performed in triplicates and shown as mean values ±SD. (TIFF) [file pone.0101521.s002.tiff]

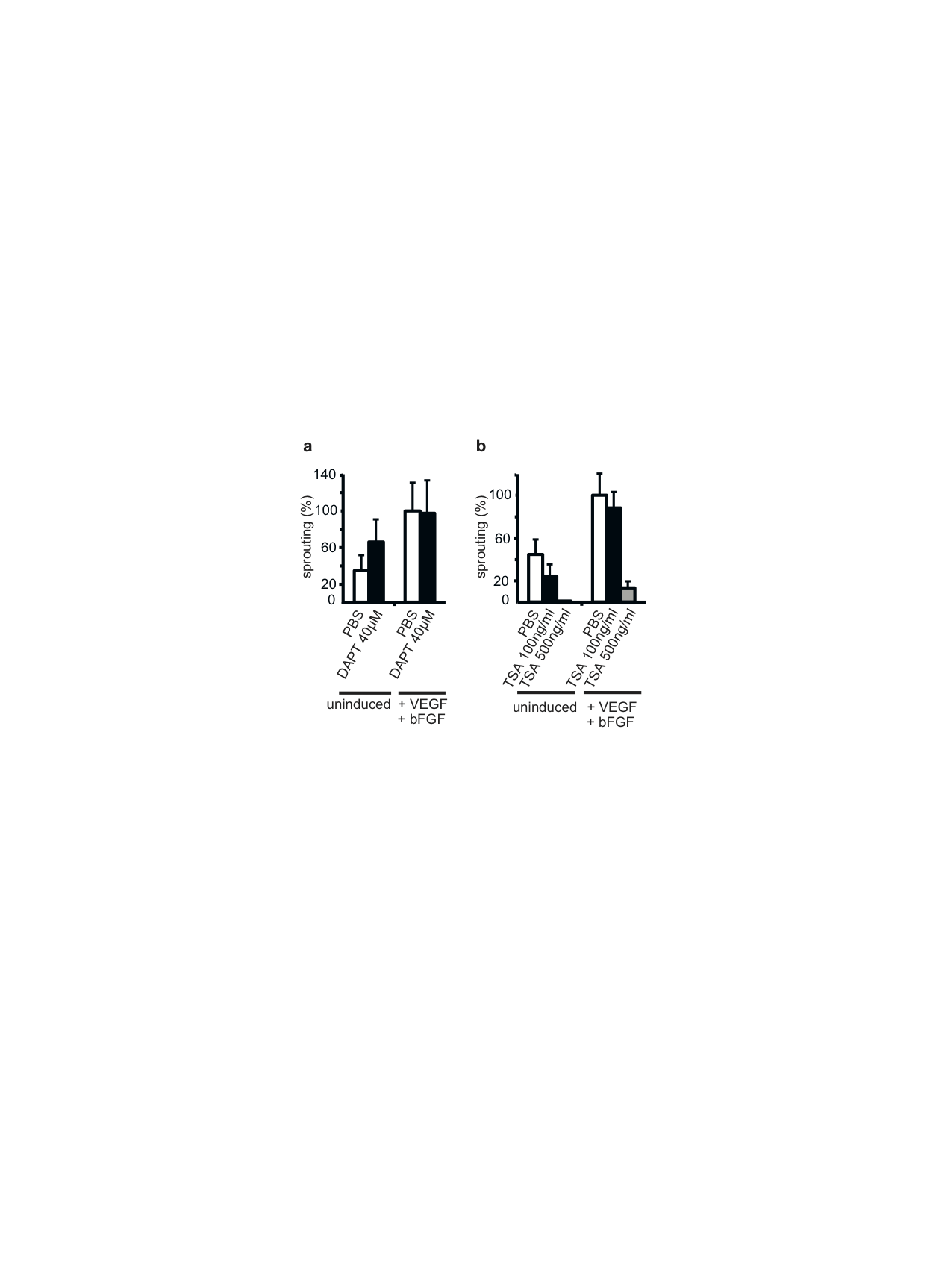

Supplement: Figure S3 — Applied concentrations of DAPT induce basal sprouting and concentrations of TSA were selected to allow sprouting without impairment of sprouting. HUVEC were tested in the spheroid sprouting assay and stimulated by VEGF and bFGF (50 ng/ml) or left uninduced. (a) Inhibition of Notch signaling by DAPT induces basal sprouting. Where indicated DAPT (40 µM) was added. (b) Effects of TSA on sprouting of HUVECs. HUVEC were tested in the presence of TSA at 100 ng/ml or 500 ng/ml concentration. Shown data are mean values ± SD and were calculated from one representative experiment out of three independent experiments performed. HUVEC induced with VEGF and bFGF but without DAPT or TSA addition were arbitrarily set to 100%. (TIFF) [file pone.0101521.s003.tiff]

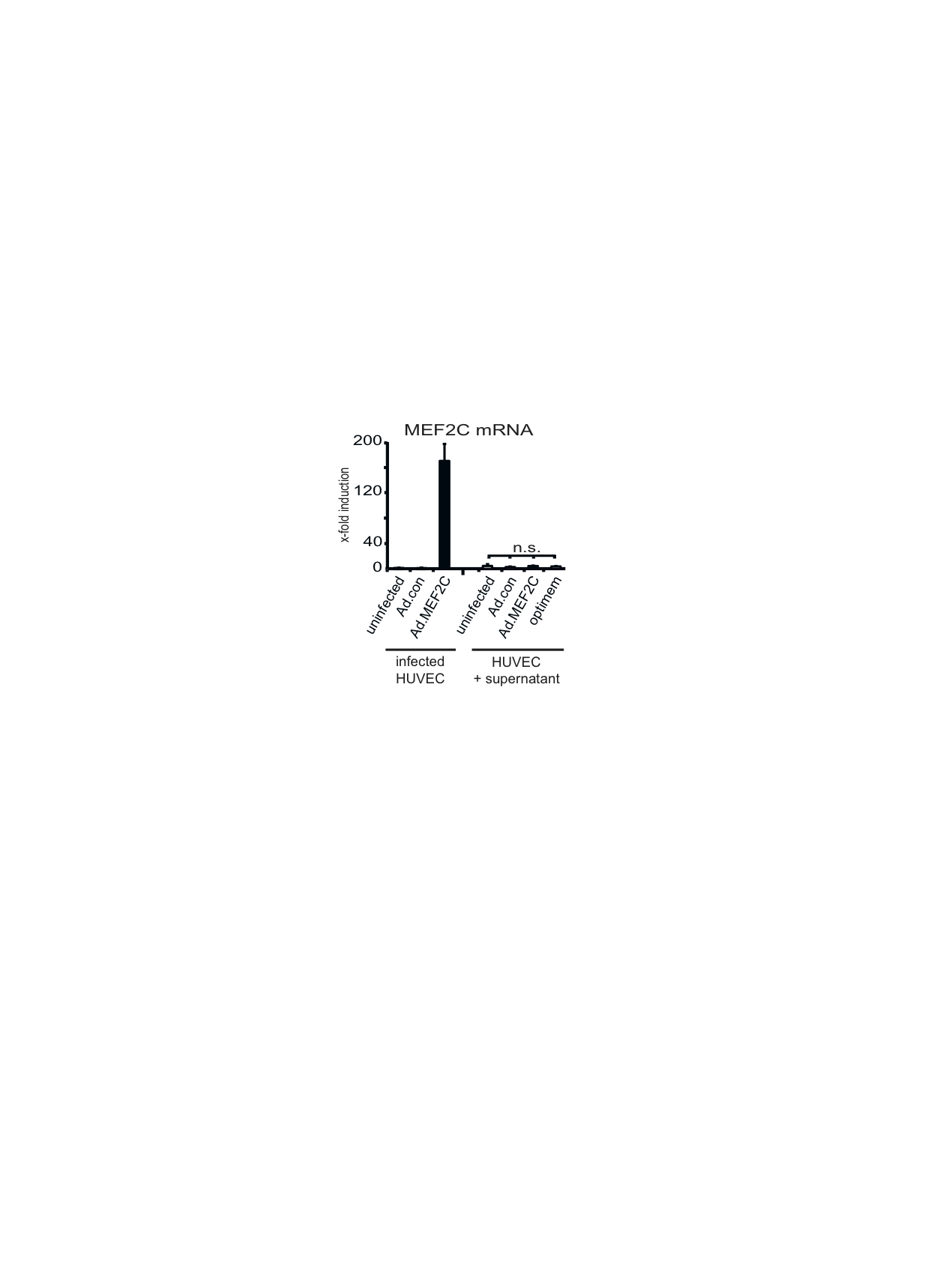

Supplement: Figure S4 — Concentrated supernatants derived from Ad.MEF2C infected cells do not contain residual adenovirus leading to overexpression of MEF2C mRNA. HUVEC were infected with Ad.MEF2C, Ad.con or left without infection for 8 hours, then medium was changed to serum-free Opti-MEM medium. Supernatants were harvested after 48 hours and further concentrated by diafiltration as described in the Methods section. Concentrated supernatants were added to monolayers of HUVECs and the cells cultured for 24 h, an equal period as used for sprout formation. Then RNA was isolated and subjected to realtime RT-PCR analysis as described in the Method section. Relative mRNA levels are shown as mean values ± SD calculated from duplicates of three independent experiments. Obtained values were normalized to beta-2-microglobulin mRNA as internal standard. (TIFF) [file pone.0101521.s004.tiff]
