# Supplementary material for: The Transcription Factor MEF2C Negatively Controls Angiogenic Sprouting of Endothelial Cells Depending on Oxygen
Source: PLoS One. 2014 Jul 2;9(7):e101521. doi: 10.1371/journal.pone.0101521 (PMC4079651; doi:10.1371/journal.pone.0101521)
Supplement: Table S1 — List of genes upregulated over three-fold by MEF2C. HUVEC were transduced with adenoviruses encoding MEF2C (Ad.MEF2C) or control adenoviruses without cDNA inserts (Ad.con) or cultured without virus transduction. 8, 16 and 32 h after transduction total RNA was isolated and subjected to microarray analysis using Affymetrix Human Gene Level 1.0 ST Gene Chips as described in the Methods section. Values for non-infected HUVEC (uninf) represent random expression intensities as measured in the microarray analysis. Changes in gene expression intensities induced in Ad.con- and Ad.MEF2C -infected cultures relative to non-infected HUVEC are displayed in the consecutive columns. (DOCX) [file pone.0101521.s005.docx]

**Supplemental Table S1**

**List of genes upregulated over three-fold by MEF2C**

HUVEC were transduced with adenoviruses encoding MEF2C (Ad.MEF2C) or control adenoviruses without cDNA inserts (Ad.con) or cultured without virus transduction. 8, 16 and 32 h after transduction total RNA was isolated and subjected to microarray analysis using Affymetrix Human Gene Level 1.0 ST Gene Chips as described in the Methods section. Values for non-infected HUVEC (uninf) represent random expression intensities as measured in the microarray analysis. Changes in gene expression intensities induced in Ad.con- and Ad.MEF2C -infected cultures relative to non-infected HUVEC are displayed in the consecutive columns.

|  | **uninf** | **Ad.con** | | | **Ad.MEF2C** | | |
| --- | --- | --- | --- | --- | --- | --- | --- |
| **Gene** | **0 h** | **8 h** | **16 h** | **32 h** | **8 h** | **16 h** | **32 h** |
| MEF2C | 558.5 | 1.01 | 0.95 | 0.77 | 12.42 | 21.96 | 29.05 |
| A2M | 85.4 | 1.12 | 0.92 | 1.08 | 0.90 | 2.97 | 11.80 |
| RAD51AP1 | 213.2 | 1.01 | 0.48 | 0.06 | 0.53 | 0.75 | 6.24 |
| SELE | 27.8 | 1.22 | 1.34 | 2.69 | 1.08 | 2.96 | 5.64 |
| STC1 | 39.0 | 1.10 | 1.06 | 1.09 | 0.89 | 1.30 | 3.81 |
| CDRT1 | 60.7 | 0.89 | 0.71 | 0.65 | 0.74 | 1.78 | 3.69 |
| LOC642533 | 116.5 | 1.20 | 0.92 | 0.48 | 0.82 | 0.76 | 3.56 |
| ADAMTS9 | 606.3 | 1.14 | 0.64 | 0.59 | 0.40 | 1.46 | 3.18 |
